# Reduction of oxidative stress in liver cancer patients by oral green tea polyphenol tablets during hepatic arterial infusion chemotherapy

**DOI:** 10.3892/etm.2012.602

**Published:** 2012-06-07

**Authors:** YASUTAKA BABA, JUN-ICHIRO SONODA, SADAO HAYASHI, NANAKO TOSUJI, SHUNRO SONODA, KANRO MAKISUMI, MASAYUKI NAKAJO

**Affiliations:** Department of Radiology, Graduate School of Medical and Dental Sciences, Kagoshima University, Sakuragaoka, Kagoshima 890-8520, Japan

**Keywords:** green tea tablet, oxidative stress, antioxidant, hepatocellular carcinoma, metastatic liver cancer, hepatic arterial infusion chemotherapy

## Abstract

Hepatic arterial infusion chemotherapy (HAI) using an implanted port system is the standard regimen for primary and metastatic liver cancers (MLCs). However, there have been few studies concerning HAI-induced oxidative stress and damage to the liver or other organs. The aim of the present study was to investigate the ability of green tea polyphenols (GTPs) to reduce the oxidative stress or increase the biological antioxidative potential in HAI-treated patients. A total of 19 patients with inoperable hepatocellular carcinoma (HCC) or MLC from colorectal malignancy were eligible for HAI with cisplatin (CDDP) and 5-fluorouracil (5FU). The study subjects were randomly assigned to either a 3 or a 6 oral GTP tablets per day group. Each tablet had a GTP content equivalent to 79 mg of epigallocatechin-3-gallate. The oxidative stress was assessed by measuring the levels of derivatives of reactive oxygen metabolites (d-ROMs) and the biological antioxidative potential (BAP) values in patient plasma using the Free Radical Analytical System 4 (FRAS4), and correlating the results with clinical laboratory data for the patients. The levels of d-ROMs were significantly reduced by the oral intake of 6 GTP tablets for 6–9 months (P=0.0463) but were not significantly reduced by the oral intake of 3 GTP tablets daily. BAP values remained constant in the 3 and 6 tablet groups for 6–9 months during the follow-up study. The total serum bilirubin (T-bil) levels increased significantly at 3 (P=0.028) and 9 (P=0.0151) months and the red blood cell (RBC) count decreased at 6 months (P=0.0458) after intake for the 6 GTP tablet group. Alkaline phosphatase (ALP) levels increased significantly at 9 months (P=0.0298). Cholinesterase (ChE) decreased significantly at 9 (P= 0.0127) and 12 (P= 0.0207) months after intake for the 3 GTP tablet group. The results indicate that the daily intake of 6 GTP tablets containing 474 mg polyphenols significantly reduces HAI-induced oxidative stress in HCC or MLC patients while the antioxidative potentials of the patients remain constant.

## Introduction

It is well known that green tea polyphenols (GTPs) have antioxidant activity (1–3) and induce apoptosis in cancer cells (4,5). These anticancer effects have been confirmed by epidemiological studies which have shown that green tea drinking lowers the risk of stomach cancer (6,7). The therapeutic merits of combining anticancer drugs with green tea have been suggested by *in vitro* and *in vivo* studies (8–11), however, to date there is no clinical evidence concerning the dose effects of green tea components on the clinical outcome. In the present study we report the first clinical investigation of the antioxidant effects of green tea in liver cancer patients by measuring two biomarkers: derivatives of reactive oxygen metabolites (d-ROMs), as an indicator of oxidative stress, and biological antioxidative potential (BAP), as an indicator of the levels of antioxidant compounds in the patients.

## Materials and methods

### Patients

From October 2009 to October 2010, a total of 19 patients with hepatocellular carcinoma (HCC) or metastatic liver cancer (MLC) from colorectal cancer were enrolled in this study. In 17 of the patients the cancer was causatively associated with chronic liver damage related to hepatitis B, hepatitis C or non-B non-C hepatitis infection and the remaining 2 patients had metastatic colon cancer (Table I). The diagnoses of HCC and MLC were made by either histopathological or radiological analysis as well by the measurement of the cancer markers serum α-fetoprotein (AFP), protein induced by vitamin K absence II or antagonist- II (PIVKA-II) and carcinoembryonic antigen (CEA). All patients had multiple cancers involving both lobes of the liver or invading the portal vein, so that they were ineligible for surgical resection and also not suitable for treatment by percutaneous radiofrequency ablation (RFA) or percutaneous ethanol injection (PEI). Thus, hepatic arterial infusion chemotherapy (HAI) was the only feasible treatment to use in these patients. The eligibility criteria for the study were as follows: Eastern Cooperative Oncology Group (ECOG) performance status 0 to 1; Child-Pugh class A or B; and adequate liver function (aminotransferase levels <5 times the normal upper limit) and renal function (creatinine levels <1.5 mg/dl). Any previous anticancer treatments, including hepatic surgery, RFA, PEI and transcatheter arterial chemoembolization (TACE), had to be terminated 8 weeks prior to the beginning of the study. Patients were excluded from the study if they had any other type of malignancy, including extrahepatic metastases, or any other serious condition.

### HAI using an implanted port system and chemotherapeutic regimen

HAI was performed using an implantable reservoir system. The method of implantation of the reservoir port system was as previously reported (12). Arteriography was performed using the Seldinger technique. After accessing the femoral artery, diagnostic arteriography was performed to evaluate the hepatic arterial and portal venous anatomy and assess the number and location of HCC or metastatic nodules with the aid of CT during hepatic arteriography and arterial portography. Following the embolization of the gastroduodenal and right gastric arteries with microcoils (Tornado, Cook Medical, Bloomington, IN, USA), a W-spiral catheter (Piolax, Kanagawa, Japan) with multiple side holes was placed at the common or proper hepatic artery under fluoroscopic guidance. The proximal end of the catheter was connected to the injection port and the device was implanted in a subcutaneous pocket in the right anterior femur. The regimen for HCC was as follows: a CDDP (cisplatin; Bristol-Myers Squibb Co., New York, NY, USA) 10 mg/0.5 h plus 5FU (5-fluorouracil; Kyowa Hakko Kirin Co., Ltd., Tokyo, Japan) 250 mg/2 h continuous infusion for 10 days as an inpatient; and then a CDDP 20 mg/0.5 h plus 5FU 250 mg/2 h infusion every 2 weeks as an outpatient. The MLC regimen was a 5FU 1,000 mg/2 h infusion every 2 weeks as an inpatient or outpatient.

### GTPs

GTP tablets were prepared from spray-dried green tea extract powder that had been processed to remove >70% of the caffeine but retain >90% of the polyphenol compounds. The extract powder was combined with 18% dextran and 2% sucrose ester and shaped into tablets. Each tablet was 9 mm in diameter, 400 mg in weight and had a GTP content equivalent to 79 mg of epigallocatechin-3-gallate (EGCg), as previously described (13).

After obtaining the approval of the Institutional Review Board and the patients’ informed consent, the study subjects were randomly assigned into one of two oral GTP tablet groups; a 3 tablets per day group (n=10) and a 6 tablets per day group (n=9). Measurements of the levels of d-ROMs, as an indicator of oxidative stress, and BAP, as an indicator of the levels of antioxidant compounds in the patients, were carried out using blood samples obtained prior to and following GTP intake and clinical laboratory data were evaluated in parallel. The patients were followed up for 12 months.

### Measurements of d-ROMs and BAP

Heparinized peripheral blood, freshly drawn from the patients, was spun in a low speed centrifuge to separate the plasma. Measurements of the d-ROMs and BAP in the plasma were carried out within 2 h using the Free Radical Analytical System 4 (FRAS4) designed by SEAC s.r.l. (Florence, Italy). For the measurement of d-ROMs, 10 *μ*l of the fresh plasma was mixed with 1 ml of a reagent containing FeCl_2_ (Fe^++^) and diethyl *p*-phenylene diamine chromogen and incubated for 5-min at 37°C to develop the oxidized color that was measured by the change in optical density at 505 nm over the 5 min reaction time. For the measurement of BAP, 1 ml of an oxyradical colorant comprising a thiocyanate compound and FeCl_3_ (Fe^+++^) was reduced by the addition of 10 *μ*l of the patient’s plasma and the decrease in optical density at 505 nm over the 5 min reaction time at 37°C was measured in the FRAS4 according to the manufacturer’s instructions (Wizmer Inc., Tokyo, Japan). d-ROM and BAP readings were taken for each sample and used to assess the level of the patient’s biological oxidative stress.

### Statistical analysis

Statistical analysis was carried out using the Student’s t-test for continuous variables and a χ^2^ test for categorical variables. The commercially available software package MedCalc Version 9.5.1.0 (MedCalc Software, Mariakerke, Belgium) was used. A two-tailed P-value <0.05 was considered to indicate a statistically significant result.

## Results

All 19 patients were followed up for 6 months, and 6 and 8 patients were lost at 9 and 12 months, respectively. The characteristics of the study subjects are presented in Table I. The degree of liver damage was classified as Child Pugh A in all patients. Previous treatments were as follows: hepatic resection (HR) alone, 3 patients; TACE alone, 5 patients; arterial infusion, 1 patient; HR plus TACE, 2 patients; HR plus TACE plus RFA, 1 patient; RFA plus TACE, 1 patient; and no treatment, 6 patients. There were statistically significant reductions in the levels of d-ROMs at 6 (P=0.0463) and 9 (P=0.0436) months after intake for the 6 GPT tablet group while no statistically significant reductions were observed in the levels of d-ROMs for the 3 tablet group (Table II). BAP values remained constant in the 3 and the 6 tablet groups, but there was a significant reduction in BAP values at 12 months (P=0.008) after intake for the 3 GTP tablet group (Table III). Total serum bilirubin (T-bil) levels significantly increased at 3 (P=0.028) and 9 (P=0.0151) months after intake for the 6 GTP tablet group while the red blood cell (RBC) count decreased at 6 months (P=0.0458) for this group (Table IV). Serum alkaline phosphatase (ALP) levels significantly increased at 9 months (P=0.0298) and cholinesterase (ChE) levels significantly decreased at 9 (P=0.0127) and 12 (P=0.0207) months after intake for the 3 GTP tablet group (Table V).

## Discussion

There are increasing numbers of studies concerning the effective synergism of GTPs with anticancer drugs to reduce drug resistance and side effects (8–10). The synergistic effect has been clearly shown in an *in vivo* mouse model of chemoresistant liver cancer in which the presence of GTPs markedly enhanced the anticancer activity of doxorubicin (DOX) by inhibiting P-glycoprotein (P-gp) efflux pump activity and enabling intracellular DOX to accumulate and effectively kill the liver cancer cells (11). The GTPs also detoxify the free radicals produced by the cancer cells by means of their inherent antioxidant activity (11). The active proliferation of cancer cells is known to cause oxidative stress by generating reactive oxygen species (ROS) and, by concomitantly modulating glutathione peroxidase (GPX) and superoxide dismutase (SOD), may lower the innate activities of free radical scavengers in cancer patients (14). Certain chemotherapeutic agents and therapeutic irradiation are known to generate ROS in cancer patients (15,16). Quiles *et al* reported that supplementation with antioxidant nutrients protected cancer patients by compromising DOX-induced oxidative damage without any loss of anticancer chemotherapeutic effects (17). It is thus conceivable that the antioxidant supplementation of cancer chemotherapy reduces oxidative stress and ameliorates the chemotherapeutic sideeffects (16,18–24). d-ROMs may be an indicator of the oxidative stress in the bodies of cancer patients since their concentration is directly proportional to the levels of active ROS and free radicals in the body (25,26). We thus successfully demonstrated that the levels of d-ROMs were significantly reduced at 6 months after intake for the 6 GTP tablet group although no significant changes in BAP values were observed during the period of our follow-up study (Tables II and III). It is likely that the local production of free radicals by cancer cells or metastatic cancer foci oxidizes lipid substances at the cancer site and leave stable d-ROMs in the body while the BAP in non-cancerous parts of the body may exceed the local levels of oxidative stress (with the exception of patients with severe conditions, including cardiovascular surgery and hemodialysis, whose BAP values are reported to be significantly affected by iatrogenic impact) (25,27). This may explain why we could not detect any significant reduction of BAP in our study subjects who had undergone moderate HAI.

Epidemiological studies have revealed that a daily intake of green tea of more than 10 cups per day lowered the incidence of cancers in the stomach, lung and other sites (28–30). In our study, there were significant reductions in the levels of d-ROMs at 6 (P=0.0463) and 9 (P=0.0436) months after intake for the 6 GTP tablet group but not the 3 GTP tablet group (Table III). The RBC count decreased and T-bil levels increased significantly at 3 and 9 months after intake for the 6 GTP tablet group (Table IV). In the 3 GTP tablet group, ChE levels decreased significantly at 9 and 12 months after intake while alkaline phosphatase (ALP) levels increased significantly at 9 months after intake (Table V), although the increments of ALP levels at 6 and 12 months showed larger variations so that they did not reach the statistical significance. The increased T-bil levels in plasma may relate to a higher turnover of GTP-bound RBCs and an enhanced oxidant-scavenging capacity in the circulation, as postulated by other investigators (31,32). It is thus likely that GTP-bound RBCs are trapped in the reticuloendothelial system for recycling of the hemoglobin moiety as a potential protector against oxidative stress. The shorter half-life of the GTP-bound erythrocytes may explain the decreased RBC counts and the increased T-bil levels in plasma observed in 6 GTP tablet group (Table IV). The 3 GTP tablet group had neither decreased RBC counts nor increased T-bil levels, indicating that a daily intake of 3 GTP tablets may be insufficient to create enough GTP-bound RBCs to protect against the oxidative stress in the HAI-treated patients. It is likely that 3 GTP tablets do not provide enough antioxidant activity to protect HAI-treated patients from liver damage, as shown by the decreased ChE and increased ALP levels in the plasma (Table V).

There are 3 limitations to this study. Firstly, this is not a randomized control study, secondly, the number of patients studied is small and thirdly, there is no direct evidence of increased antioxidant stress during the cancer treatment process. In order to address these limitations, we have initiated a fully randomized control trial studying two new patient groups, one taking GTP tablets and the other taking placebo tablets, which are followed up by the same protocol as is used in the current study.

This is the first study concerning the quantitative evaluation of HAI-induced oxidative stress in patients with liver cancer. This harmful oxidative stress may be prevented by supplementation with oral GTP tablets.

## Figures and Tables

**Table I:** Profile of the hepatocellular carcinoma and metastatic liver cancer patients supplemented with oral GTP tablets.

Characteristics	3 GTP tablet group (n=10)	6 GTP tablet group (n=9)
Age (years), median (range)	75.5 (61–82)	68 (49–76)
Gender (male/female)	6/4	7/2
Hepatitis virus		
None	0	2
B	2	0
C	6	5
Non-B non-C	2	2
Child-Pugh score (median)	6	6
Child-Pugh class A	10	9
Treatments prior to supplementation		
None	2	4
Surgery	2	1
TACE	3	2
AI	0	1
Surgery + TACE	1	1
Surgery + TACE + RFA	1	0
RFA + TACE	1	0

TACE, transcatheter arterial chemoembolization; AI, arterial infusion; RFA, radiofrequency ablation; GTP, green tea polyphenol.

**Table II:** Reduction in d-ROMs in the plasma of the patients following the intake of oral GTP tablets.

Dose	Follow-up					P-value
	Before	3 months	6 months	9 months	12 months	
6 tablet group	n=9	n=9	n=9	n=6	n=5	
	430.5 (74.6)	401.5 (64.4)	376.7 (60.0)^a^	348.8 (59.2)^b^	365.4 (57.7)^c^	0.0463^a^, 0.0436^b^
3 tablet group	n=10	n=10	n=10	n=7	n=6	
	391.8 (87.4)	397.9 (130.2)	410.5 (151.1)	373.8 (90.9)	377.3 (100.3)	NS

All numbers are the average d-ROM readings and the standard deviation (SD). NS, not statistically significant.

a Significant difference in d-ROM readings taken before and 6 months after the intake of 6 GTP tablets.

b Significant difference in d-ROM readings taken before and 9 months after the intake of 6 GTP tablets.

c Not yet analyzed due to the small number of subjects (n=5). d-ROMs, derivatives of reactive oxygen metabolites; GTP, green tea polyphenol. The units for d-ROM are Carratelli units (Carr units).

**Table III:** Change in BAP in the plasma of the patients following the intake of oral GTP tablets.

Dose	Follow-up					P-value
	Before	3 months	6 months	9 months	12 months	
6 tablet group	n=9	n=9	n=9	n=6	n=5	
	2653.1 (368.8)	2540.1 (175.2)	2482.3 (211.6)	2483.0 (148.7)	2627.6 (297.2)^a^	NS
3 tablet group	n=10	n=10	n=10	n=7	n=6	
	2694.8 (230.0)	2526 (160.5)	2639.6 (165.9)	2652.8 (263.1)	2590.6 (158.9)^b^	0.0078^b^

All numbers are the average BAP readings and the standard deviation (SD).

a Not yet analyzed due to the small number of patients (n=5).

b Significant difference in BAP readings taken before and 12 months after the intake of 3 GTP tablets. NS, not statistically significant; BAP, biological antioxidative potential; GTP, green tea polyphenol. The units for BAP are micromole/l.

**Table IV:** Clinical laboratory data of the 6 GTP tablet group.

	Follow up					P-value
	Before	3 months	6 months	9 months	12 months^a^	
PIVKA II (mAU/ml)	2,664.40 (5,796.7)	1,285.50 (2,540.0)	3,473.50 (8,318.9)	172.00 (268.9)	3,403.00 (6,686.8)	NS
WBC (/*μ*l)	3,932.20 (1,476.5)	3,872.20 (1,691.5)	3,902.20 (1,249.8)	4,476.00 (2,218.9)	3,685.00 (1,432.6)	NS
RBC (×10^4^/*μ*l)	399.30 (60.8)	394.40 (55.6)	374.10 (66.4)^b^	382.50 (77.9)	394.60 (89.0)	0.0458^b^
Hb (g/dl)	12.60 (1.9)	12.90 (1.5)	12.40 (1.8)	12.90 (2.0)	13.10 (2.2)	NS
Plt (×10^4^/*μ*l)	13.10 (5.1)	11.70 (4.8)	10.90 (4.1)	10.20 (3.8)	11.30 (2.9)	NS
AST (IU/l)	52.60 (29.7)	62.70 (57.2)	69.70 (45.4)	57.30 (39.0)	64.20 (54.3)	NS
ALT (IU/l)	51.20 (37.1)	55.90 (55.1)	52.90 (32.3)	46.80 (28.3)	52.20 (45.9)	NS
LDH (IU/l)	226.00 (74.2)	273.60 (144.6)	247.60 (58.8)	220.20 (84.8)	228.40 (40.5)	NS
ALP (IU/l)	353.90 (79.4)	411.90 (204.4)	398.90 (152.9)	438.30 (260.3)	403.40 (169.3)	NS
ChE (IU/l)	210.40 (83.2)	211.60 (86.2)	200.00 (109.5)	234.50 (138.8)	202.20 (118.1)	NS
T-bil (mg/dl)	0.62 (0.22)	0.84 (0.15)^b^	0.74 (0.30)	0.88 (0.26)^c^	0.94 (0.29)	0.028^b^, 0.0151^c^
BUN (mg/dl)	12.00 (2.4)	14.40 (6.1)	14.20 (5.4)	13.70 (3.2)	12.60 (5.3)	NS
Cr (mg/dl)	0.70 (0.1)	0.70 (0.1)	0.70 (0.2)	0.70 (0.2)	0.70 (0.3)	NS
CRP (mg/dl)	0.70 (1.1)	0.80 (1.8)	0.30 (0.3)	0.50 (0.6)	1.00 (1.9)	NS
AFP (ng/ml)	9,193.90 (2,2651.0)	9,709.00 (22,515.3)	9,427.40 (22,577.3)	1,845.90 (4,083.2)	1,330.80 (2,650.8)	NS
Collagen IV (ng/ml)	234.40 (91.0)	261.20 (89.6)	304.60 (102.7)	269.50 (68.1)	252.00 (83.5)	NS

All numbers are listed as average and standard deviation (SD). NS, not statistically significant difference.

a Not yet analyzed due to small number of patients (n=5).

b,c Significant difference compared with before treatment. GTP, green tea polyphenol; PIVKA-II, protein induced by vitamin K absence II or antagonist II; WBC, white blood cell; RBC, red blood cell; Hb, hemoglobin; Plt, platelets; AST, aspartate transaminase; ALT, alanine transaminase; LDH, lactate dehydrogenase; ALP, alkaline phosphatase; ChE, cholinesterase; T-bil, total serum bilirubin; BUN, blood urea nitrogen; Cr, creatinine; CRP, C-reactive protein; AFP, α-fetoprotein.

**Table V:** Clinical laboratory data of the 3 GTP tablet group.

	Follow up					P-value
	Before	3 months	6 months	9 months	12 months	
PIVKA II (mAU/ml)	1,136.70 (1,876.0)	1,260.00 (1,955.9)	5,918.90 (15,296.1)	3,367.00 (4,446.7)	3,630.80 (5,251.8)	NS
WBC (/*μ*l)	4,137.00 (3,129.2)	2,979.00 (764.6)	3,147.00 (1,331.8)	2,785.70 (990.0)	2,895.00 (1,139.2)	NS
RBC (×10^4^/*μ*l)	380.70 (48.2)	379.20 (46.9)	378.30 (33.1)	367.00 (36.1)	368.30 (46.4)	NS
Hb (g/dl)	13.00 (1.0)	13.00 (1.2)	13.00 (0.9)	12.50 (1.2)	12.50 (1.1)	NS
Plt (×10^4^/*μ*l)	9.60 (1.8)	9.10 (2.3)	9.20 (3.4)	9.60 (4.1)	11.30 (7.2)	NS
AST (IU/l)	47.10 (16.6)	55.50 (21.5)	53.70 (27.5)	55.70 (19.4)	56.80 (29.5)	NS
ALT (IU/l)	35.90 (11.8)	40.90 (22.9)	41.60 (17.9)	42.10 (17.9)	41.70 (24.2)	NS
LDH (IU/l)	244.40 (65.4)	296.90 (119.7)	300.80 (141.1)	241.90 (60.4)	227.80 (44.6)	NS
ALP (IU/l)	366.00 (156.9)	392.00 (171.2)	527.70 (450.1)	414.70 (120.5)^a^	427.80 (121.3)	0.0298^a^
ChE (IU/l)	182.30 (40.6)	185.30 (47.3)	167.90 (56.7)	143.30 (45.6)^a^	143.70 (54.3)^b^	0.0127^a^, 0.0207^b^
T-bil (mg/dl)	0.81 (0.24)	0.75 (0.24)	0.88 (0.20)	0.87 (0.26)	1.02 (0.60)	NS
BUN (mg/dl)	15.10 (4.1)	15.40 (5.7)	22.70 (21.4)	15.40 (6.2)	15.20 (4.8)	NS
Cr (mg/dl)	0.80 (0.2)	0.74 (0.17)	0.90 (0.5)	0.80 (0.2)	0.80 (0.2)	NS
CRP (mg/dl)	0.50 (0.5)	0.50 (0.7)	0.40 (0.5)	1.00 (1.0)	0.60 (0.4)	NS
AFP (ng/ml)	783.40 (1,940.6)	760.90 (1,946.0)	3,439.90 (7,990.5)	8,728.60 (22,829.5)	581.70 (751.1)	NS
Collagen IV (ng/ml)	311.20 (57.4)	319.40 (55.9)	349.40 (132.1)	336.40 (64.9)	326.80 (68.0)	NS

All numbers are listed as average and standard deviation (SD). NS, no statistically significant difference.

a,b Significant difference compared with before treatment. GTP, green tea polyphenol; PIVKA-II, protein induced by vitamin K absence II or antagonist II; WBC, white blood cell; RBC, red blood cell; Hb, hemoglobin; Plt, platelets; AST, aspartate transaminase; ALT, alanine transaminase; LDH, lactate dehydrogenase; ALP, alkaline phosphatase; ChE, cholinesterase; T-bil, total serum bilirubin; BUN, blood urea nitrogen; Cr, creatinine; CRP, C-reactive protein; AFP, α-fetoprotein.
